# Therapy optimization in metastatic colorectal cancer guided by discordant KRAS mutation status between primary and metachronous recurrent lesions: a precision oncology case report

**DOI:** 10.1097/RC9.0000000000000382

**Published:** 2026-03-30

**Authors:** Hiroshi Shintani, Katsuji Tokuhara, Naoki Kataoka

**Affiliations:** Department of Surgery, Kishiwada Tokushukai Hospital, Kishiwada, Japan

**Keywords:** anti-EGFR therapy, bevacizumab, case report, genetic heterogeneity, molecular profiling, precision oncology

## Abstract

**Introduction:**

In stage IV colorectal cancer, molecularly targeted therapies such as anti-EGFR and anti-VEGF antibodies improve patient outcomes. Anti-EGFR therapy is typically effective in RAS wild-type tumors, whereas RAS mutations confer resistance and necessitate alternative strategies. However, discrepancies in RAS status between primary and metastatic lesions may influence treatment response.

**Presentation of Case:**

We report a 50-year-old male who underwent resection for rectal adenocarcinoma initially characterized as RAS wild-type. He subsequently developed liver metastases and was treated with FOLFIRI plus panitumumab. While some metastatic lesions regressed, others progressed. Molecular analysis of the liver metastasis revealed a KRAS mutation, in contrast to the primary tumor. The regimen was switched to FOLFIRI plus bevacizumab, which achieved marked regression of all metastatic lesions.

**Discussion:**

This case underscores the clinical significance of intertumoral heterogeneity in RAS status. The emergence of a KRAS mutation in the metastatic site explained resistance to anti-EGFR therapy and highlighted the importance of reassessing tumor genetics during disease progression. Tailoring treatment to the molecular profile of the metastatic lesion led to improved clinical outcomes.

**Conclusion:**

In colorectal cancer, repeat genetic testing of metastatic lesions should be considered, particularly in cases of treatment resistance. Molecular reassessment may reveal actionable alterations that guide therapy selection and optimize patient management.

## Introduction

Systemic chemotherapy remains the cornerstone of treatment for stage IV colorectal cancer^[^1,2^]^, and the integration of molecularly targeted agents has further improved clinical outcomes^[^3,4^]^. For patients with left-sided colorectal cancer, anti-EGFR antibodies are recommended in the absence of RAS mutations, whereas the presence of RAS mutations renders these agents ineffective due to downstream activation of the EGFR signaling pathway^[^5^]^. In such cases, bevacizumab in combination with chemotherapy is typically preferred^[^6^]^. Although RAS mutation status is usually concordant between primary and metastatic colorectal tumors, rare cases of discordance have been reported^[^7,8^]^.

**HIGHLIGHTS**

- RAS mutation discordance occurred between primary rectal tumor and liver metastasis.
- Therapy was modified from FOLFIRI + panitumumab to FOLFIRI + bevacizumab.
- Treatment modification resulted in significant regression of liver metastases.
- Genetic heterogeneity between primary and metastatic colorectal cancer can impact therapeutic choice.
- Reassessment of tumor molecular status is recommended when initial therapy fails.

This case report describes a patient with discordant RAS mutation status between the primary and metastatic tumors, where adjustments of targeted therapy resulted in clinical improvement. This work has been reported in line with the SCARE 2025 criteria^[^9^]^.

## Presentation of case

**Figure 1.**
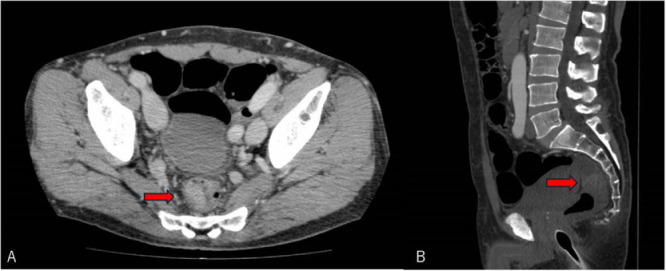
Preoperative contrast-enhanced CT images demonstrating the primary rectal tumor. (A) Axial contrast-enhanced CT showing a contrast-enhancing mass in the middle rectum (arrow). (B) Sagittal contrast-enhanced CT showing the same rectal tumor (arrow).

A 50-year-old male presented with progressive anemia and a positive fecal occult blood test. Colonoscopy identified a rectal tumor in the middle rectum. The exact distance from the anal verge was not documented. Serum carcinoembryonic antigen (CEA) level was within the normal range (3.7 ng/mL). Preoperative contrast-enhanced CT of the chest, abdomen, and pelvis revealed a contrast-enhancing mass in the middle rectum, with no evidence of distant metastatic lesions (Fig. 1). Preoperative pelvic MRI was not performed. The clinical stage was cT3N0M0. Neoadjuvant chemoradiotherapy was not administered, as it is not routinely recommended for upper rectal tumors in the Japanese clinical guidelines, and upfront surgical resection was selected. In September 2022, he underwent laparoscopic low anterior resection of the rectum. Histopathological examination demonstrated subserosal invasion (pT3) with metastases in two of 18 regional lymph nodes (pN1b), corresponding to stage IIIb disease according to the UICC/AJCC 8th edition. Surgical margins were negative (R0 resection). RAS mutation analysis was conducted using a PCR–reverse sequence-specific oligonucleotide (PCR-rSSO) assay, and no RAS mutations were detected in the resected specimen. Microsatellite instability testing was negative, whereas mismatch repair immunohistochemistry was not performed.

The patient received adjuvant chemotherapy with CAPOX (capecitabine plus oxaliplatin) for 6 months (eight cycles). Capecitabine was administered orally at 1000 mg/m^2^ twice daily on days 1–14, and oxaliplatin was given intravenously at 130 mg/m^2^ on day 1 of each 21-day cycle. Treatment was well tolerated apart from grade 1 peripheral neuropathy, and all planned cycles were completed. Surveillance imaging and tumor marker monitoring showed no evidence of recurrence. In August 2023, contrast-enhanced CT revealed a solitary 28-mm lesion in liver segment S8, consistent with metachronous liver metastasis (distant recurrence) from rectal cancer. PET-CT demonstrated an SUVmax of 6.88. The patient underwent laparoscopic partial hepatectomy with curative intent for this metachronous liver metastasis. The resected specimen measured 50 × 35 mm and was positive for SATB2 on immunostaining, confirming colorectal origin. At the time of surgery, the serum CEA level remained within the normal range (2.0 ng/mL).

In anticipation of recurrence, systemic chemotherapy with FOLFIRI plus panitumumab was initiated in October 2023. The regimen consisted of irinotecan 150 mg/m^2^, 5-fluorouracil 400 mg/m^2^ as a bolus followed by 2400 mg/m^2^ as a 46-h continuous infusion, and panitumumab 6 mg/kg, administered every 14 days. Although the rash was manageable, treatment was discontinued after the first cycle due to a grade 2 rash at the patient’s strong request. The patient was subsequently monitored with regular blood tests and imaging, with plans to restart therapy if recurrence was confirmed.

**Figure 2.**
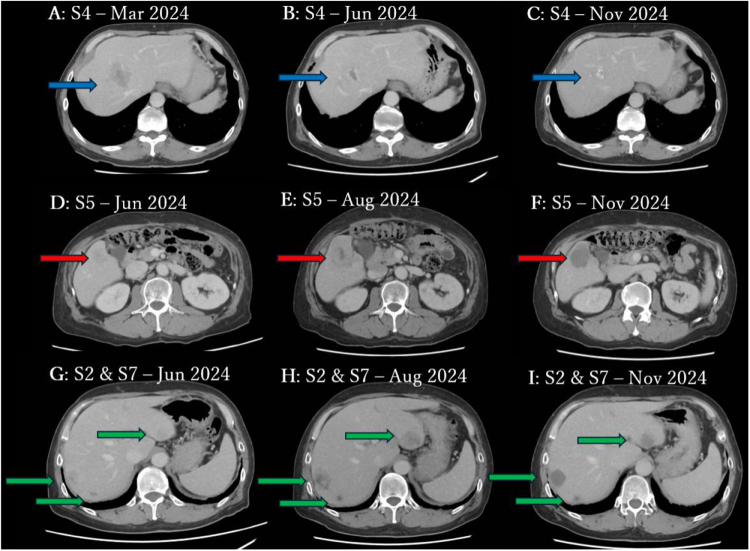
Serial contrast-enhanced CT images demonstrating differential responses of liver metastases to panitumumab- and bevacizumab-based chemotherapy, corresponding to the lesions listed in Table 1. (A–C) Lesion L3 located in liver segment 4 (S4) is shown in March 2024 (A), June 2024 (B), and November 2024 (C) (blue arrows). This lesion showed tumor shrinkage during panitumumab-based therapy, which was further maintained after switching to bevacizumab, accompanied by loss of contrast enhancement. (D–F) Lesion L5 located in segment 5 (S5) is shown in June 2024 (D), August 2024 (E), and November 2024 (F) (red arrows). This lesion progressed with enlargement and persistent contrast enhancement during panitumumab-based therapy, but markedly regressed with disappearance of enhancement after switching to bevacizumab. (G–I) Lesions L1 (segment 2) and L6/L7 (segment 7) are shown in June 2024 (G), August 2024 (H), and November 2024 (I) (green arrows). These lesions similarly demonstrated progression during panitumumab-based therapy, followed by significant regression and loss of contrast enhancement after initiation of bevacizumab. Lesion numbering corresponds to that used in Table 1.

In March 2024, the patient’s serum CEA level increased to 67.9 ng/mL. Contrast-enhanced CT revealed new liver lesions measuring 46 mm in segment S4, 15 mm in S5, and 19 mm in S7, all with venous-phase enhancement (Fig. 2, Table 1). A 6-mm nodule was also detected in the lower lobe of the right lung, suggestive of distant metastases from rectal cancer. Based on these findings, disease recurrence was confirmed, and systemic chemotherapy was restarted. On 6 April 2024, FOLFIRI plus panitumumab was administered with dose adjustments (irinotecan 120 mg/m^2^, 5-fluorouracil 300 mg/m^2^ bolus followed by 2000 mg/m^2^ as a 46-h continuous infusion, and panitumumab 6 mg/kg, administered every 14 days). After five cycles, a June 2024 CT scan showed regression of the S4 lesion (from 46 mm to 15 mm) and the S7 lesion (from 19 mm to 12 mm), both losing contrast enhancement. In contrast, the S5 lesion enlarged to 38 mm, and new lesions were identified in segments S2 (23 mm), S3 (26 mm), S4 (17 mm), and S7 (26 mm), indicating disease progression. Given the heterogeneous response to panitumumab, molecular analysis using next-generation sequencing (NGS) was retrospectively performed on the liver metastasis specimen resected in August 2023, which revealed a KRAS Q61H mutation. A summary of molecular testing results is provided in Supplemental Digital Content Table 1, available at: http://links.lww.com/IJSCR/A24. Despite continuing FOLFIRI plus panitumumab for 10 cycles, only the S4 and S7 lesions continued to regress, while the remaining lesions progressed with persistent enhancement. The overall condition was assessed as progressive disease. In August 2024, in light of the KRAS mutation and lack of panitumumab efficacy, treatment was switched to FOLFIRI plus bevacizumab (irinotecan 150 mg/m^2^, 5-fluorouracil 400 mg/m^2^ bolus, 2400 mg/m^2^ continuous infusion, and bevacizumab 5 mg/kg every 14 days). After six cycles, a November 2024 CT scan demonstrated a reduction in all target lesions, all of which exhibited poor contrast enhancement, suggestive of necrosis. No new lesions were identified.

**Table 1:** Serial radiologic changes of individual liver metastases during systemic therapy.

Lesion ID	Liver segment	Mar-24	Jun-24	Aug-24	Nov-24
L1	S2	ND	23 mm(+)	39 mm(+)	28 mm(−)
L2	S3	ND	26 mm(+)	37 mm(+)	22 mm(−)
L3	S4	46 mm(+)	15 mm(−)	13 mm(−)	13 mm(−)
L4	S4	ND	17 mm(+)	30 mm(+)	22 mm(−)
L5	S5	15 mm(+)	38 mm(+)	60 mm(+)	46 mm(−)
L6	S7	19 mm(+)	12 mm(−)	9 mm(−)	7 mm(−)
L7	S7	ND	26 mm(+)	47 mm(+)	30 mm(−)

The table summarizes the longitudinal changes in size and contrast enhancement of individual liver metastases as assessed by contrast-enhanced computed tomography. Lesions are numbered sequentially (L1–L7). Liver segments are defined according to the Couinaud classification. Values represent the maximum tumor diameter (mm). “(+)” indicates the presence of contrast enhancement in the venous phase, and “(−)” indicates the absence of enhancement. “ND” indicates lesions that were not detected at the corresponding time point. Lesions 3 and 4 represent two distinct metastatic lesions located within segment 4 of the liver, and lesions 6 and 7 represent two distinct metastatic lesions located within segment 7. Systemic chemotherapy consisted of FOLFIRI plus panitumumab from October 2023 to August 2024, followed by FOLFIRI plus bevacizumab from August 2024 onward.

As of the most recent evaluation, the patient has received 21 cycles of FOLFIRI plus bevacizumab. The overall response has been classified as a partial response. Serial imaging has demonstrated continued shrinkage of the liver metastases, whereas the pulmonary metastases have remained stable in size. Treatment is ongoing. A summary of the clinical timeline is provided in Table 2. A summary of systemic treatment regimens is provided in Supplemental Digital Content Table 2, available at: http://links.lww.com/IJSCR/A25.

**Table 2:** Timeline of key clinical events, including diagnosis, surgical interventions, systemic treatments, molecular testing, and follow-up. The table summarizes the sequence of disease recurrence, treatment modifications based on molecular findings, and therapeutic responses during the clinical course.

Date	Event	Details
September 2022	Diagnosis and primary surgery	Laparoscopic low anterior resection for rectal cancer (pT3N1bM0, Stage IIIb)
September 2022	Molecular testing (primary tumor)	RAS mutation analysis showed wild-type status
October 2022–March 2023	Adjuvant chemotherapy	CapeOX for 6 months (8 cycles)
August 2023	Metachronous liver metastasis detected	Solitary 28-mm lesion in segment S8 on contrast-enhanced CT and PET-CT
August 2023	Liver resection	Laparoscopic partial hepatectomy with curative intent
October 2023	Systemic therapy initiated	FOLFIRI plus panitumumab (Cycle 1)
October 2023	Treatment interruption	Discontinued after one cycle due to grade 2 rash at patient’s request
March 2024	Disease recurrence	Rising CEA and new liver and lung metastases on CT
April 2024–August 2024	Re-challenge with anti-EGFR therapy	FOLFIRI plus panitumumab (dose-adjusted)
June 2024	Heterogeneous treatment response	Some lesions regressed, others progressed
July 2024	Molecular testing (metastatic lesion)	Retrospective analysis of resected liver metastasis revealed KRAS Q61H mutation
August 2024	Treatment modification	Switched to FOLFIRI plus bevacizumab
November 2024	Treatment response	Marked regression of all liver metastases on CT
Most recent follow-up	Ongoing treatment	Partial response maintained after 21 cycles of FOLFIRI plus bevacizumab

CapeOX, capecitabine plus oxaliplatin; CEA, carcinoembryonic antigen; CT, computed tomography; FOLFIRI, irinotecan, 5-fluorouracil, and leucovorin.

## Discussion

This case illustrates a rare scenario in which the RAS mutation differed between the primary rectal cancer and its metastatic counterpart. While the primary tumor was RAS wild-type, a KRAS mutation was later identified in the liver metastasis upon disease progression. This discrepancy led to a shift in systemic therapy from FOLFIRI plus panitumumab to FOLFIRI plus bevacizumab, which achieved a favorable response. The case highlights the importance of repeat molecular profiling in treatment-resistant disease to guide optimal therapy selection.

In general, for patients with left-sided colorectal cancer, regimens incorporating anti-EGFR antibodies such as panitumumab or cetuximab are recommended in the absence of RAS mutations^[^1,2,6^]^. In contrast, when RAS mutations are present, anti-EGFR therapy has limited efficacy^[^5^]^.

In the present case, FOLFIRI plus panitumumab was initially selected based on the RAS wild-type status of the primary rectal tumor. However, due to an inconsistent treatment response, further molecular testing was performed on the resected liver metastasis (S8), which revealed a RAS mutation. On this basis, therapy was switched to FOLFIRI plus bevacizumab, resulting in marked regression of the liver metastases. This clinical course underscores the limited efficacy of panitumumab in RAS-mutated tumors and highlights the therapeutic benefit of tailoring treatment to the molecular characteristics of metastatic lesions. In colorectal cancer, RAS mutation is usually highly concordant between primary and metastatic tumors, and testing of either specimen is often considered sufficient^[^10^]^. Nonetheless, discrepancies may occur in treatment-resistant cases. Knijn *et al* reported one such case among 305 patients, where the primary tumor was KRAS wild-type, while the liver metastasis harbored a mutation^[^7^]^. Similarly, Fujiyoshi *et al* documented two discrepancies among 557 cases^[^8^]^. Although these events are rare, they emphasize the clinical value of repeat molecular profiling in metastatic tissue when unexpected resistance to therapy arises.

Several mechanisms may account for discrepancies in RAS mutation status between primary and metastatic colorectal cancer lesions. One factor is the variability in detection sensitivity, which can be influenced by tumor cell content and biopsy site^[^11^]^. Another possibility is the acquisition of new genetic alterations during disease progression. Compared with primary tumors, metastatic lesions are more likely to harbor additional mutations and often exhibit lower intratumoral heterogeneity, suggesting that molecular information derived from metastatic sites may be more reliable for guiding therapy^[^12^]^. Such discordance may also reflect clonal selection during metastatic progression, whereby minor subclones with growth advantage expand preferentially at metastatic sites. Although low-frequency RAS-mutant clones in the primary tumor could theoretically escape detection with conventional assays and later become dominant in metastases, the RAS Q61H mutation identified in this case is not typically regarded as a low-frequency alteration. Therefore, clonal evolution during metastatic progression is considered a more plausible explanation in the present case.

In this case, among multiple liver metastases, some lesions responded to panitumumab, while others progressed, suggesting that distinct genetic profiles may have existed across different metastatic sites. However, molecular analysis was performed on only a single resected metastatic lesion; therefore, intrametastatic or intermetastatic genetic heterogeneity could not be comprehensively assessed in this case. Previous studies have also indicated that discordance in RAS mutation status between primary and metastatic tumors is associated with poorer prognosis^[^13^]^, particularly when unexpected resistance to targeted therapy is encountered.

Based on the present case, repeat molecular testing should be considered when disease progression is observed despite anti-EGFR-based therapy, particularly at the time of newly detected or progressing metastatic lesions. Whenever feasible, analysis of tissue from the progressing metastatic site may provide the most clinically relevant information. Several practical barriers may limit the implementation of repeat molecular testing in clinical practice. First, access to next-generation sequencing may be restricted due to institutional availability, cost, or turnaround time. In such situations, alternative molecular assays with lower technical requirements may be considered to reassess key driver mutations. Second, tissue sampling from metastatic lesions may not always be feasible, particularly in cases of unresectable disease or when biopsy poses a high procedural risk. In these settings, circulating tumor DNA analysis may serve as a less invasive alternative for detecting emerging genetic alterations. Furthermore, when resistance to anti-EGFR therapy is clinically evident, switching to anti-VEGF–based therapy without additional molecular testing may represent a reasonable and pragmatic treatment strategy.

Even when the primary tumor appears controlled by chemotherapy, progression of metastatic lesions should prompt consideration of underlying genetic differences. This case underscores the importance of repeat molecular testing when resistance to first-line therapy is observed. Such reassessment can enable timely adjustment of treatment and may be valuable not only in colorectal cancer but also across other malignancies where targeted therapy plays a central role.

This case has several limitations. First, molecular analysis was performed on only a single resected metastatic lesion; therefore, intratumoral and intermetastatic genetic heterogeneity could not be fully evaluated. Second, circulating tumor DNA (ctDNA) analysis was not performed, which might have provided additional insights into clonal evolution and the genetic landscape of multiple metastatic lesions. Future studies incorporating multi-region tissue sampling and liquid biopsy may better elucidate tumor heterogeneity and guide precision treatment strategies.

## Patient perspective

The patient expressed satisfaction that the change in treatment led to an improvement in his condition and was grateful for the individualized treatment approach.

## Conclusion

This case highlights a rare instance in which the primary rectal cancer was RAS wild-type, while a recurrent liver metastasis harbored a RAS mutation. As tumors evolve, genetic alterations may differ between primary and metastatic sites. Clinicians should be aware of these potential discrepancies, particularly in cases of treatment resistance or disease progression, and consider molecular testing of metastatic lesions to guide personalized therapy.

## Data Availability

The data that support the findings of this study are available from the corresponding author upon reasonable request.
